# Genetically Predicted Causal Relationship Between Polycystic Ovary Syndrome and Preeclampsia

**DOI:** 10.1155/ije/5536687

**Published:** 2026-07-14

**Authors:** Guige Xue, Xuegui Zhou, Huan Zhang, Jinfang Wang, Xiaohua Song

**Affiliations:** ^1^ Department of Obstetrics and Gynecology, Binzhou People’s Hospital, Binzhou, Shandong, China, bzrmyy.com.cn

**Keywords:** causal relationship, genomewide association study, Mendelian randomization study, polycystic ovary syndrome, preeclampsia

## Abstract

**Background:**

Clarifying the causal relationship between polycystic ovary syndrome (PCOS) and its characteristic markers with preeclampsia (PE) is of utmost importance in preventing the development of PE. Observational studies have investigated the relationship, but the conclusions have been inconsistent.

**Methods:**

Utilizing publicly accessible genomewide association study (GWAS) data, we performed a two‐sample Mendelian randomization analysis to investigate causal relationships between PCOS, its characteristic biomarkers, and PE. The inverse variance weighting (IVW) method served as the primary analytical approach. To ensure methodological rigor, we assessed potential pleiotropy using the MR‐Egger intercept and evaluated heterogeneity through Cochran’s Q test with associated *p* values.

**Results:**

The study’s results showed a causal relationship between PCOS and PE (IVW OR = 1.172, 95% CI: 1.003–1.371, *p* = 0.046). Using the IVW method, we also discovered that characteristic indicators of PCOS, including decreased high‐density lipoprotein (HDL) levels, increased body mass index (BMI), and elevated fasting insulin levels, pose a risk for PE. At the same time, there is no clear causal relationship between low‐density lipoprotein (LDL), total cholesterol (TC), triglycerides (TGs), testosterone (T), fasting blood glucose (FBG), and circulating sex hormone–binding globulin (SHBG) and PE.

**Conclusion:**

Our research results provide new evidence at the genetic prediction level for PCOS as a risk factor for PE. Furthermore, analysis of PCOS characteristics suggests that HDL levels, BMI, and fasting insulin levels may be causally associated with PE. These findings provide a novel theoretical basis and practical strategies for the clinical management of women with PCOS, aimed at predicting and preventing PE.

## 1. Introduction

Preeclampsia (PE) is a common and serious pregnancy complication, with a global incidence of 3%–5% [1], and is a significant contributor to maternal and neonatal mortality [2]. Currently, apart from terminating pregnancy and delivering the fetus and placenta, there are no other effective treatment options for PE. Therefore, early prediction and prevention of PE are significant for improving maternal and fetal outcomes. However, the currently known risk factors are still not sufficiently accurate in predicting its occurrence. Therefore, identifying more reliable risk factors is crucial for early identification and prediction of PE.

Polycystic ovary syndrome (PCOS) is a female endocrine disorder characterized by an unknown etiology and a highly heterogeneous clinical presentation, affecting approximately 11%–13% of women [3]. It is characterized by excess androgens, menstrual irregularities, and polycystic ovaries [4]. The Rotterdam criteria were established as the most commonly employed diagnostic guidelines for identifying PCOS. Research has found many similarities in pathological changes between PCOS and PE. Clinical features commonly observed in PCOS patients, including obesity, lipid metabolism abnormalities, insulin resistance, hyperandrogenism, endothelial dysfunction, and increased chronic inflammation, all contribute to an increased risk of PE [5–9]. The “Recommendations from the 2023 International Evidence–based Guideline for the Assessment and Management of PCOS” recommends considering PCOS as a high‐risk condition during pregnancy [3], and some scholars have suggested incorporating PCOS as a risk factor into obstetric guidelines for PE [10]. However, the “WHO recommendations on antiplatelet agents for the prevention of PE” do not include PCOS as a risk factor for PE [11].

Several observational studies have analyzed the relationship between PCOS and PE [12–16]. A meta‐analysis revealed that the risk of developing PE is increased by 30% in women with PCOS compared to those without the syndrome [12]. Another nationwide registry‐based cohort study also indicated a 29% increased risk of developing PE in women with PCOS [10]. However, a retrospective case–control study arrived at an opposite conclusion, suggesting that the increased risk of PE in the case group compared to the control group was not directly related to the history of PCOS but possibly attributed to features associated with PCOS, such as obesity, insulin resistance, hyperandrogenism, and lipid abnormalities [13]. Discrepancies among studies may stem from racial/geographic variations in PCOS manifestations, leading to differential risks of pregnancy complications across PCOS phenotypes. The causal relationship between PCOS and PE remains inconclusive, possibly due to confounding factors and reverse causation in observational studies.

Mendelian randomization (MR) studies employ genetic variants as instrumental variables (IVs) to analyze causal relationships between exposures and outcomes; genetic variants are randomly allocated during meiosis, which can mitigate the influence of confounding factors and reverse causation present in traditional observational studies [17, 18]. In addition, utilizing summary statistics from genomewide association study (GWAS) significantly enhances the causal inference capability of two‐sample MR analyses [19]. Previous MR studies have investigated the causal relationships between PCOS and various conditions, such as Guan et al. finding no causal relationship between PCOS and gestational diabetes mellitus (GDM), but establishing a causal association between PCOS characteristic indicators and GDM [20]; Cao et al. analyzing the causal association between PCOS and different subtypes of breast cancer [21]; and Zhu et al. concluding that PCOS itself does not increase the risk of Type 2 diabetes, coronary heart disease, and stroke, but rather emphasizes the high‐risk characteristics of PCOS [22].

To date, no MR study has unveiled the causal relationship between PCOS and its characteristic indicators with PE. Therefore, in this study, we utilized a two‐sample MR analysis to investigate the potential causal relationship between PCOS and PE at the genetic prediction level and to identify which pathological features of PCOS are associated with the occurrence of PE.

## 2. Materials and Methods

### 2.1. Study Design

**FIGURE 1:**
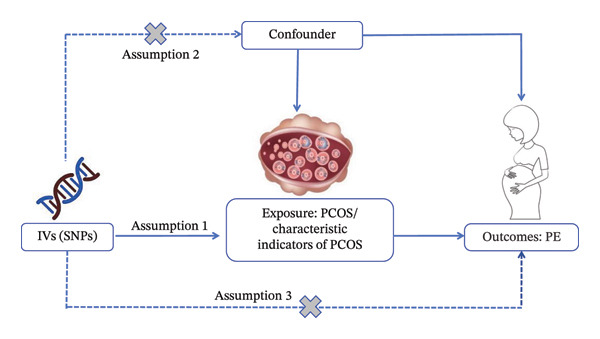
Flowchart of the two‐sample MR study design.

This study employed two‐sample MR analysis, using PCOS and its characteristic indicators as exposure factors, single‐nucleotide polymorphisms (SNPs) significantly associated with the exposure as IVs, with the outcome being PE. MR analysis must adhere to three core assumptions [18]; (1) the relevance assumption: a strong correlation exists between genetic variants and the exposure factor; (2) the independence assumption: genetic variants are independent of confounding factors that influence the exposure and outcome; (3) the exclusion–restriction assumption: genetic variants only affect the outcome through the exposure and not through other pathways (Figure 1).

### 2.2. Data Sources

The genetic variant data related to PCOS were obtained from the latest and most comprehensive published GWAS meta‐analysis, including 10,074 PCOS patients and 103,164 healthy control individuals. The diagnostic criteria were based on the National Institutes of Health (NIH) (2540 cases and 15,020 controls) or Rotterdam criteria (2669 cases and 17,035 controls), or self‐reported diagnosis (5184 cases and 82,759 controls) [23]. The genetic variant data related to PE were sourced from the Finn Biobank, comprising 4255 cases and 114,735 controls. All SNPs were derived from European populations. The GWAS ID information for PCOS characteristic indicators can be found in Supporting Table 1.

### 2.3. Selection of IVs

The criteria for selecting IVs were as follows: independent SNPs (*r*2 < 0.001 and kb > 10,000 kb), *p* value < 5 × 10 − 8. The F‐statistic for all included SNPs was greater than 10 [24, 25]. We identified 14 genetic variants from the PCOS GWAS database and ultimately included 12 SNPs as IVs for the analysis of the causal association between PCOS and PE after harmonizing the exposure–outcome dataset (Supporting Table 2).

### 2.4. MR Analysis

In this study, the inverse variance weighting (IVW) method was mainly used to assess the causal relationship between PCOS and its characteristic indicators with PE, using genetic variance as an IV. The results were expressed as odds ratios (ORs) and 95% confidence intervals (CIs) and were set to be statistically significant at *p* < 0.05. To improve the reliability of our MR analysis results, heterogeneity was assessed using Cochran’s Q test and its *p* value, and horizontal pleiotropy was assessed using the MR‐Egger intercept. Funnel plots were created to identify potential outlier SNPs, scatter plots, forest plots, and leave‐one‐out plots to visualize the MR results. All analyses were performed using the R software (Version 4.3.3) and the two‐sample MR package.

## 3. Results

### 3.1. Causal Association Between PCOS and PE

**FIGURE 2:**
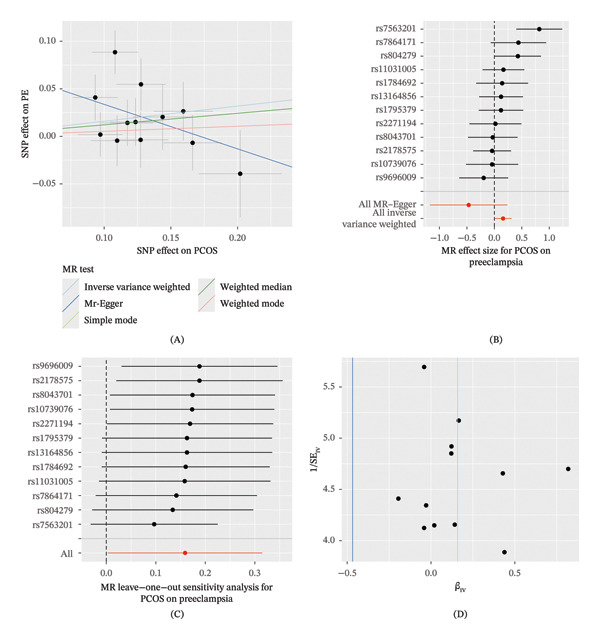
Mendelian randomization estimates of the causal effect of PCOS on PE. (A) Scatter plot of SNP effects on PCOS against SNP effects on PE. (B) Forest plot of individual SNP causal estimates. (C) Leave‐one‐out sensitivity analysis. (D) Funnel plot for assessment of overall heterogeneity and symmetry.

The results of MR analysis under the IVW method showed that PCOS was a risk factor for PE (IVW OR = 1.172, 95% CI: 1.003–1.371, *p* = 0.046) (see Table 1). Regarding heterogeneity testing, the MR‐Egger Q value was 13.571, *p* = 0.193; IVW Q value was 17.853, *p* = 0.085, indicating no heterogeneity. A further test for pleiotropy was performed, and the MR‐Egger intercept was 0.0804, *p* = 0.106, *p* > 0.05, indicating that there was no pleiotropy. Figure 2 shows the scatter plot, MR causality plot, leave‐one‐out sensitivity analysis plot, and funnel plot of PCOS and PE. The sensitivity analysis of leave‐one‐out showed no significant outlier SNPs. The funnel plot exhibited approximate symmetry, indicating no apparent heterogeneity.

**TABLE 1:** Causal association between PCOS and PE.

Methods	IVs (n SNPs)	b	Se	pval	OR	95% CI
Inverse variance weighted	12	0.159	0.079	0.045	1.172	1.002, 1.370
MR‐Egger	12	−0.467	0.360	0.223	0.626	0.309, 1.269
Weighted median	12	0.121	0.081	0.136	1.128	0.962, 1.324
Simple mode	12	0.053	0.130	0.690	1.054	0.817, 1.360
Weighted mode	12	0.053	0.123	0.674	1.054	0.828, 1.342

*Note:* PCOS, polycystic ovary syndrome; PE, preeclampsia; n, number of SNPs.

Abbreviations: IV, instrumental variable; SNP, single‐nucleotide polymorphism.

### 3.2. The Causal Relationship Between Various Characteristic Indicators of PCOS and PE: Based on the IVW Method

**FIGURE 3:**
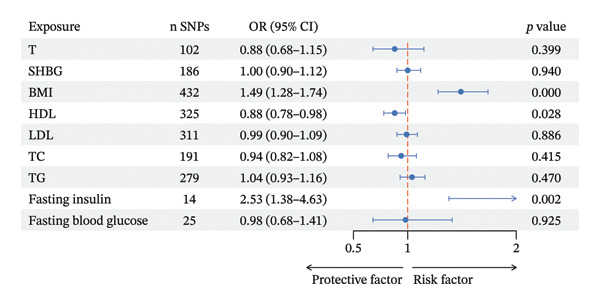
Forest plot of the associations between genetically predicted polycystic ovary syndrome (PCOS)–related traits and the risk of preeclampsia (PE). Estimates are presented as odds ratios (ORs) with 95% confidence intervals (CIs). HDL: high‐density lipoprotein; LDL: low‐density lipoprotein; BMI: body mass index; FBG: fasting blood glucose; T: testosterone; SHBG: sex hormone–binding globulin; TG: triglyceride; TC: total cholesterol.

The IVW method showed a causal relationship between high‐density lipoprotein (HDL), fasting insulin, BMI and PE. A decrease in HDL levels increases the risk of PE (OR = 0.88, 95% CI = 0.79–0.99, *p* = 0.029); an increase in fasting insulin levels and BMI is associated with an increased risk of PE (OR = 2.54, 95% CI: 1.39–4.63, *p* = 0.003 and OR = 1.50, 95% CI: 1.29–1.75, *p* = 0.000). However, there is no causal relationship between low‐density lipoprotein (LDL), total cholesterol (TC), triglyceride (TG), testosterone (T), fasting blood glucose (FBG), and sex hormone–binding globulin (SHBG) with PE (Figure 3). The results of the heterogeneity and multiplicity analyses are summarized in Supporting Table 3. Supporting Figures 1–4 include scatter plots, forest plots, leave‐one‐out plots, and funnel plots.

## 4. Discussion

The aim of our study was to explore the potential causal relationship between PCOS and PE at the genetic prediction level using existing large‐scale GWAS data, as well as to identify which pathological features of PCOS are associated with the occurrence of PE. The results of this two‐sample MR study indicate a clear causal association between genetically predicted PCOS and a higher risk of PE, suggesting that women with PCOS may be at increased risk of PE. Further analysis of PCOS characteristic indicators revealed that only a decrease in HDL levels among lipid indicators is significantly causally associated with an increased risk of PE, while other lipid metabolism indicators, including TC, TG, and LDL, show no significant correlation with PE at the genetic level. In addition, we found that an increase in BMI and fasting insulin levels is associated with an increased risk of PE, whereas there is no clear causal relationship between T, FBG, and SHBG and PE. The reliability of the MR results was further confirmed by Cochran’s Q heterogeneity test and leave‐one‐out sensitivity analysis.

Previous observational studies have mostly indicated that PCOS is a risk factor for PE, but there are different conclusions; Joshi et al. showed that the increased risk of PE in patients with PCOS is not related to the PCOS disease per se, but may be associated with some metabolic characteristics of PCOS [13]. The inconsistency in the findings of observational studies may be related to the fact that the clinical presentation of PCOS is highly heterogeneous, and the degree of perinatal complications in patients with PCOS varies according to ethnicity and geography [4]. This study primarily utilized the IVW method for MR analysis, which concluded that PCOS is a risk factor for PE. The Cochran’s Q heterogeneity test and leave‐one‐out sensitivity analysis further validated the reliability of the MR results. This research provides new evidence for PCOS as a risk factor for PE.

The prevalence of abnormal lipid metabolism in patients with PCOS can be as high as 70%, characterized by reduced HDL levels and increased cholesterol [26]. The “PCOS” recommends screening for blood lipids in PCOS patients but does not specify which lipid indicator holds greater diagnostic significance [4]. Therefore, our study included all four lipid indicators—HDL, LDL, TC, and TG—in the MR analysis using the IVW method. The results showed a significant causal relationship between decreased HDL levels and an increased risk of PE, while no causal relationship was found between LDL, TC, TG, and PE. HDL exerts multiple protective effects on the vascular endothelium. First, HDL promotes the generation of nitric oxide (NO) by stimulating endothelial NO synthase (eNOS) via a Sphingosine‐1‐phosphate (S1P)–dependent pathway, thereby maintaining endothelial function and vasodilation [27, 28]. Second, HDL possesses intrinsic anti‐inflammatory and antioxidant properties: it inhibits the expression of adhesion molecules on endothelial cells and reduces oxidative stress by preventing LDL oxidation [29]. Third, HDL participates in lipid metabolism regulation by facilitating reverse cholesterol transport and preventing lipid peroxidation damage [30]. However, in PE, HDL becomes dysfunctional beyond merely quantitative reduction. Studies have shown that HDL isolated from women with PE loses its antioxidant capacity and is depleted of S1P and Apolipoprotein M (apoM), which are critical for NO production, thereby contributing to hypertension and proteinuria [31]. Based on these findings, we recommend that preconception and prenatal care for PCOS women should prioritize the evaluation of blood lipids, particularly HDL levels. The results can guide lifestyle adjustments based on the findings and, when necessary, increase the frequency of antenatal check‐ups to prevent the occurrence of PE.

The most common metabolic characteristics of PCOS are insulin resistance and compensatory hyperinsulinemia, affecting approximately 50%–80% of PCOS patients [32, 33]. Currently, the widely used method for assessing insulin resistance in clinical practice is the Homeostasis Model Assessment of Insulin Resistance (HOMA‐IR) index, calculated as follows: HOMA‐IR = fasting insulin (μU/dL) × FBG (mmol/L)/22.5 [34]. This study included the two indicators, fasting insulin and FBG, involved in the calculation formula for MR causal analysis. The results indicated that an increase in fasting insulin levels is a risk factor for PE. Hyperinsulinemia activates the sympathetic nervous system and upregulates Endothelin‐1 receptor expression, leading to increased blood pressure. In addition, hyperinsulinemia induces hypertriglyceridemia, which promotes endothelial dysfunction and reduces prostacyclin production [35]. These mechanisms, together with increased oxidative stress and chronic inflammation, create a prohypertensive and proinflammatory environment that increases susceptibility to PE.

In addition, insulin resistance in PCOS patients is also associated with hyperandrogenism. Both insulin resistance and hyperinsulinemia can directly stimulate ovarian tissue to produce androgens and can indirectly promote hyperandrogenism by inhibiting the synthesis of SHBG [36, 37]. Several observational studies have shown that women with PCOS and higher levels of T during pregnancy seem to have a higher risk of PE, which is related to the ability of T to interfere with trophoblast invasion as well as placental morphology and function [7, 38, 39].

However, the MR analysis in this study suggests that T and SHBG are not associated with the risk of PE, which is contrary to the results of previous observational studies, and more in‐depth studies are needed to explore the relationship between the two in terms of pathological mechanisms.

Obesity is a common comorbidity of PCOS, affecting 20%–80% of PCOS patients [40]. Obesity exacerbates the endocrine abnormalities in patients with PCOS and increases the incidence of the condition [41]. Excessive adipose tissue secretes proinflammatory adipokines (e.g., leptin and resistin) while reducing anti‐inflammatory adiponectin, creating a systemic inflammatory milieu that impairs endothelial function and promotes hypertension [42, 43]. Moreover, obesity‐associated hyperlipidemia and oxidative stress contribute to placental ischemia and the release of antiangiogenic factors, hallmarks of PE pathogenesis [44]. The MR results in this study confirmed at the genetic prediction level that BMI is a risk factor for PE. Therefore, it is recommended that all PCOS women take appropriate measures to manage their weight.

Our study has several main strengths. First, the use of MR as a research methodology, the use of genetic variation as an IV, the fulfillment of chronological plausibility, and the avoidance of confounding factors and reverse causality as much as possible. Second, our GWAS data were all from European ancestry, avoiding ethnic heterogeneity. Finally, the genetic data for our exposure and outcome are from different cohorts, enhancing the credibility of the results.

Nevertheless, this study also has certain limitations. First, the study cannot rule out unidentified potential confounding factors, which may lead to violations of the second and third assumptions, thus affecting the reliability of the results. Second, the clinical manifestations of PCOS exhibit regional and racial differences, while the participants in the GWAS data used in this article are all of European ancestry; therefore, the applicability of our results to other ethnicities remains to be verified. Third, we found that the detection of HDL among the lipid markers was more meaningful in predicting PE, but this is a genetic prediction conclusion, and we will further validate this conclusion in the next step by collecting clinical data from women with PCOS in a comparative analysis according to the presence or absence of concomitant PE.

## 5. Conclusion

In conclusion, this two‐sample MR study provides new evidence for a causal relationship between PCOS and PE. In addition, analysis of PCOS characteristics suggests that HDL levels, BMI, and fasting insulin levels may be causally associated with PE. These findings provide new rationale and ideas for managing women with PCOS to predict and prevent the occurrence of PE.

## Funding

No funding was received for this study.

## Conflicts of Interest

The authors declare no conflicts of interest.

## Supporting Information

Additional supporting information can be found online in the Supporting Information section.

## Supporting information


**Supporting Information 1** Supporting Table 1: Summary of GWAS data sources for PCOS and its related traits used in the two‐sample MR analysis.

**Supporting Information 2** Supporting Table 2: Detailed information of the IVs used in the MR analysis (exposure: PCOS; outcome: PE).

**Supporting Information 3** Supporting Table 3: Results of heterogeneity and pleiotropy tests for the MR analyses.

**Supporting Information 4** Supporting Figure 1. Scatter plots for the causal associations between PCOS‐related traits and PE.

**Supporting Information 5** Supporting Figure 2. Forest plots for the causal associations between PCOS‐related traits and PE.

**Supporting Information 6** Supporting Figure 3. Leave‐one‐out plots for the causal associations between PCOS‐related traits and PE.

**Supporting Information 7** Supporting Figure 4. Funnel plots for the causal associations between PCOS‐related traits and PE.

## Data Availability

The data that support the findings of this study are available from the corresponding author upon reasonable request.
