# Supplementary material for: Genetically Predicted Causal Relationship Between Polycystic Ovary Syndrome and Preeclampsia
Source: Int J Endocrinol. 2026 Jul 14;2026:5536687. doi: 10.1155/ije/5536687 (PMC13366495; doi:10.1155/ije/5536687)
Supplement: Supplementary file 4 — Supporting Information 4 Supporting Figure 1. Scatter plots for the causal associations between PCOS‐related traits and PE. [file IJE-2026-5536687-s004.pdf]

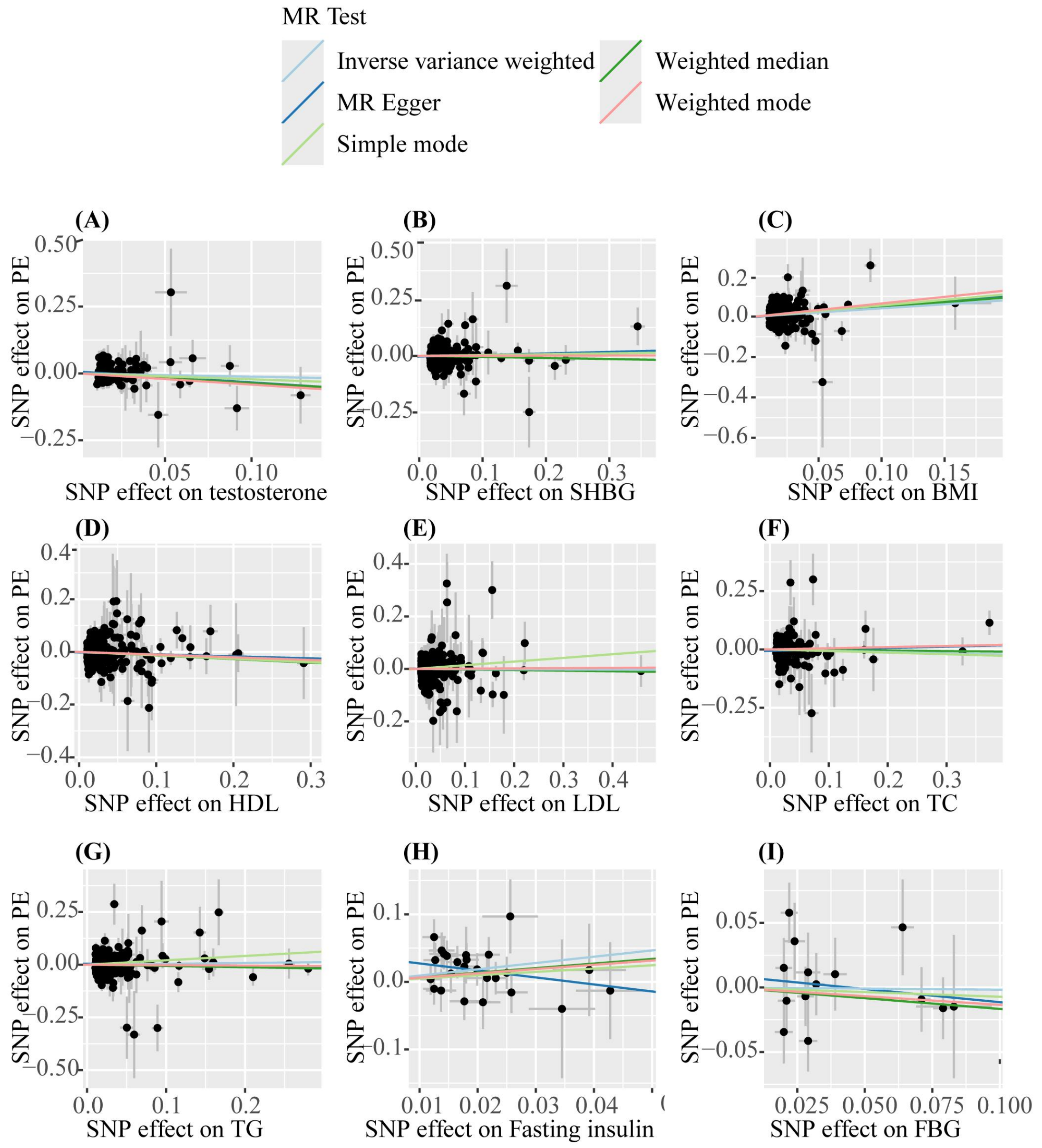

Supplementary Figures 1. Scatter plots for the association of PCOS characteristic indicators associated SNPs with risk of PE. (A) T, (B) SHBG, (C) BMI ,(D) HDL,(E) LDL,(F) TC,(G) TG,(H) Fasting insulin,(I) Fasting blood glucose.HDL,high-density lipoprotein;LDL,Low density lipoprotein;BMI,body mass index;T, testosterone; SHBG, sex hormone binding globulin;TG,Triglyceride;TC,total cholesterol;PCOS Polycystic ovary syndrome; PE, preeclampsia; SNP, single-nucleotide polymorphism.
