# Supplementary material for: Genetically Predicted Causal Relationship Between Polycystic Ovary Syndrome and Preeclampsia
Source: Int J Endocrinol. 2026 Jul 14;2026:5536687. doi: 10.1155/ije/5536687 (PMC13366495; doi:10.1155/ije/5536687)
Supplement: Supplementary file 5 — Supporting Information 5 Supporting Figure 2. Forest plots for the causal associations between PCOS‐related traits and PE. [file IJE-2026-5536687-s005.pdf]

(A)

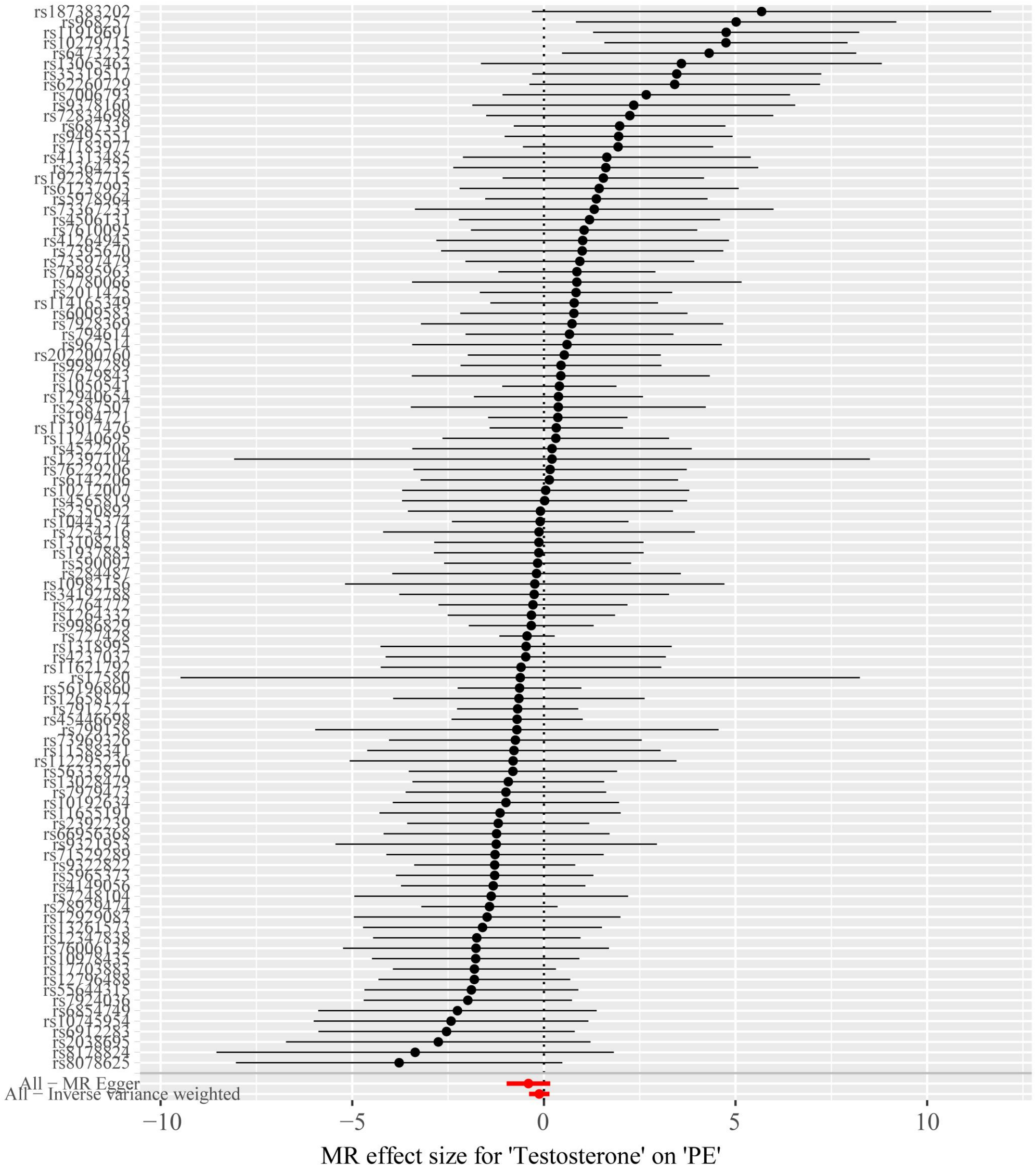
rs187383202
rs8078625
All – MR Egger
All – Inverse variance weighted
−10
−5
0
5
10
MR effect size for 'Testosterone' on 'PE'

(B)

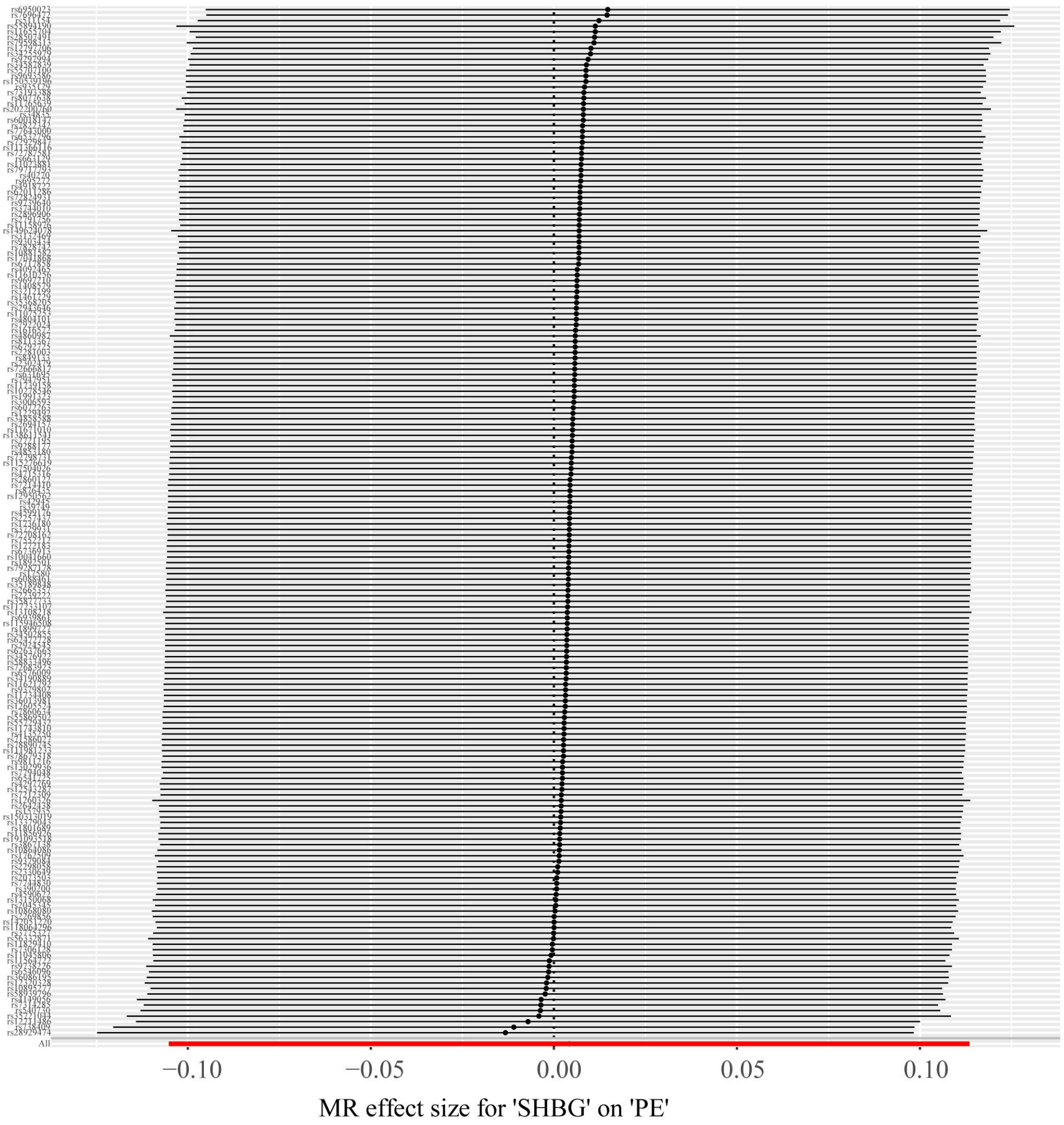

(C)

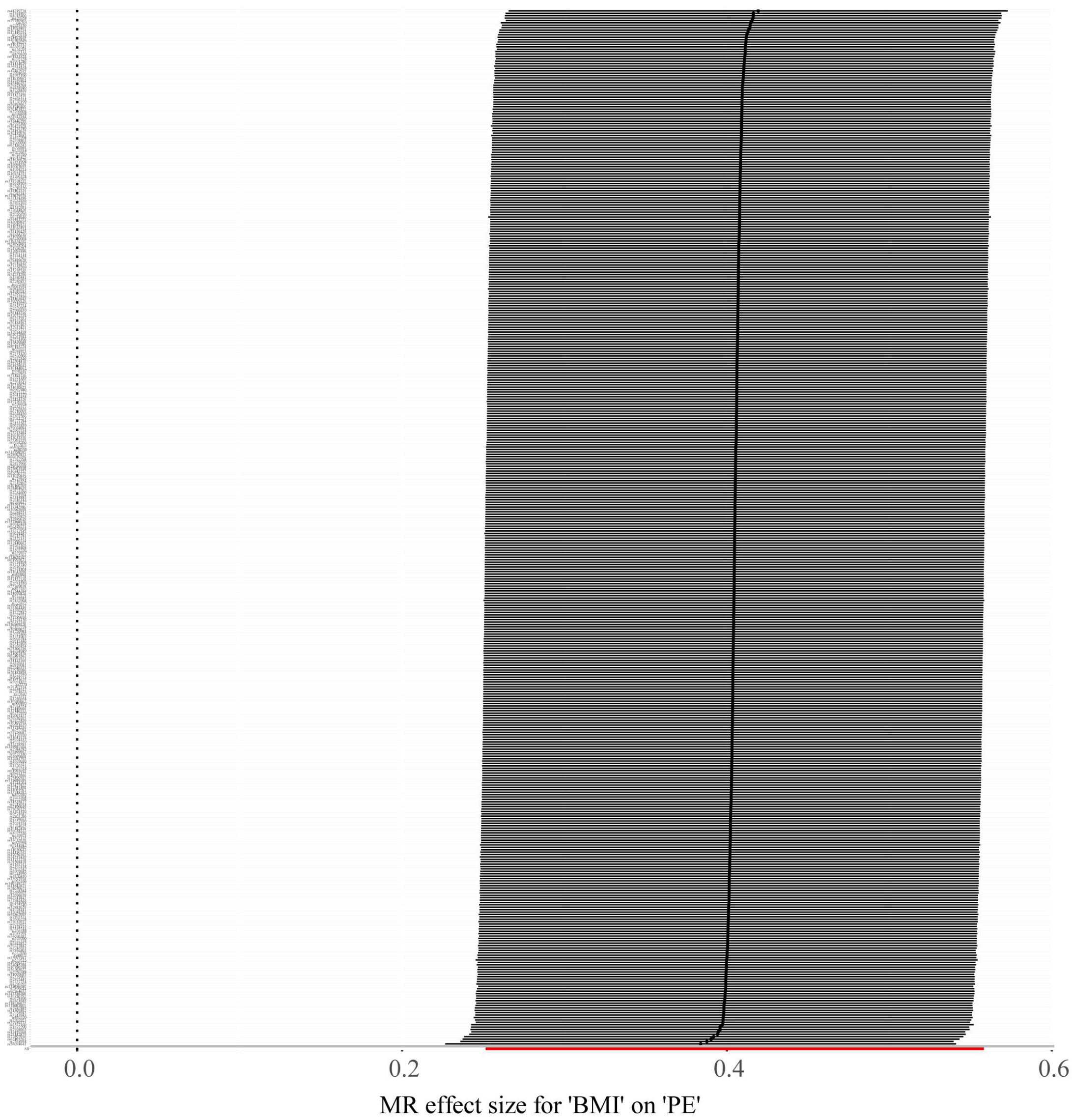

(D)

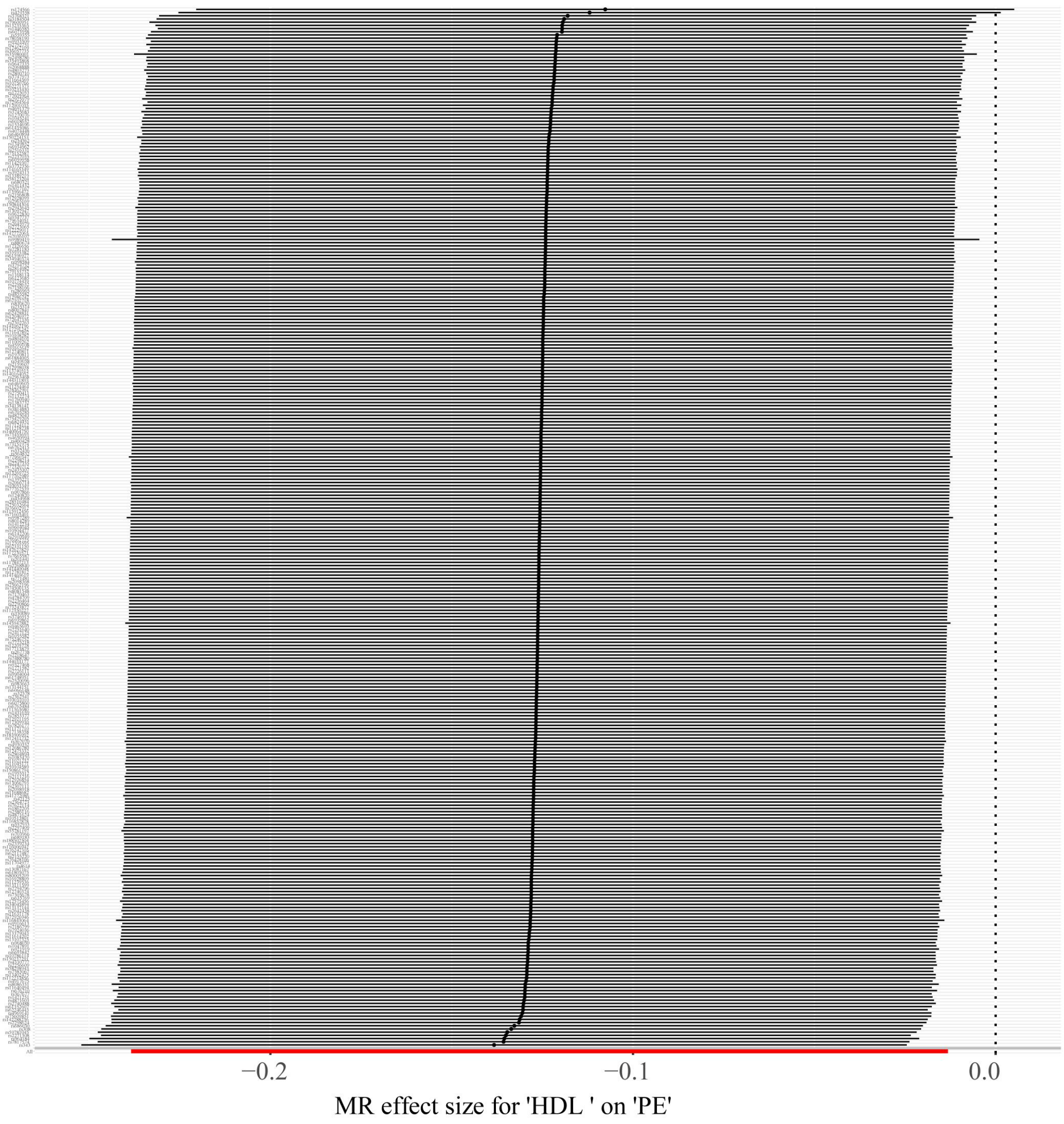

(E)

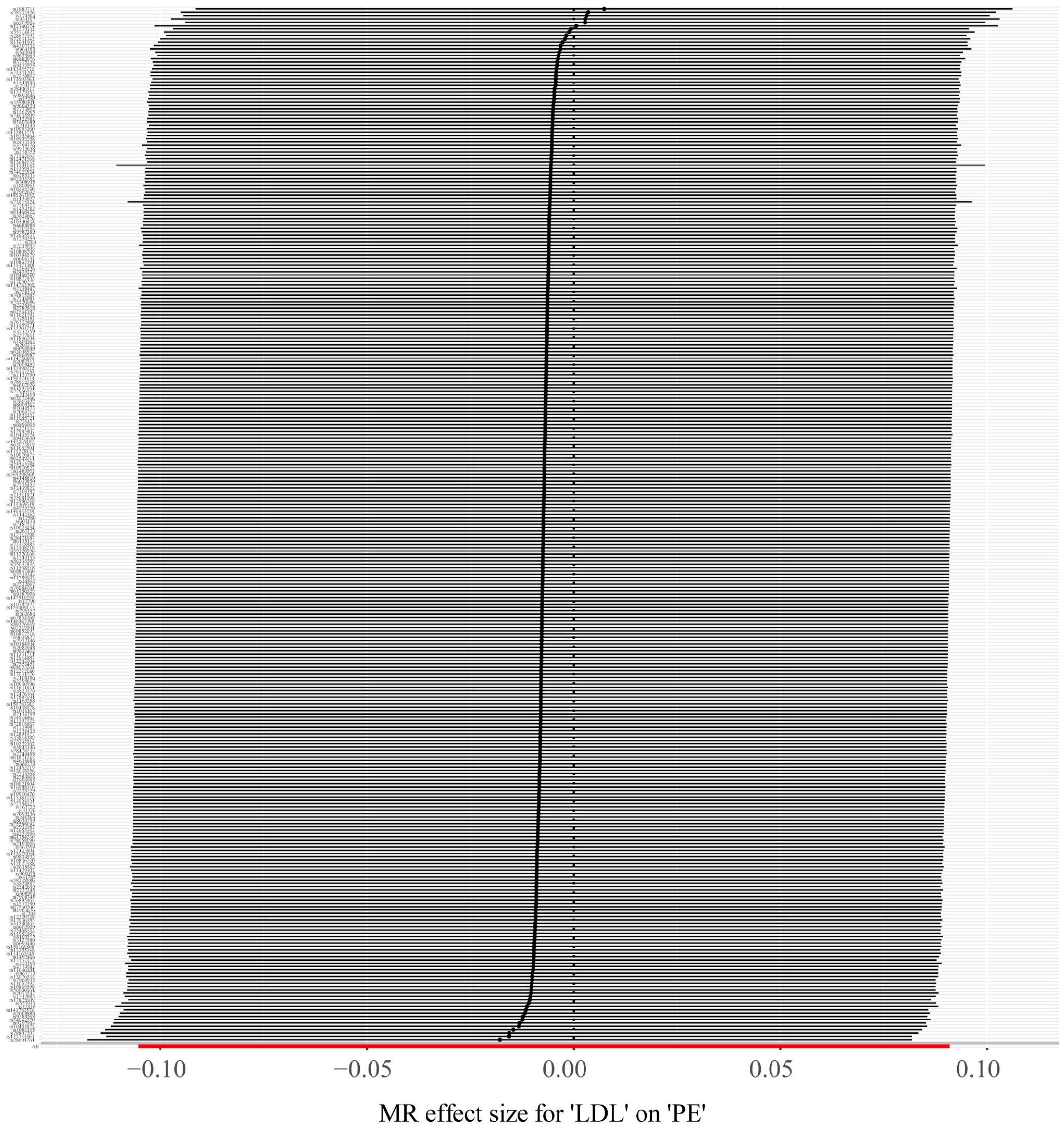

(F)

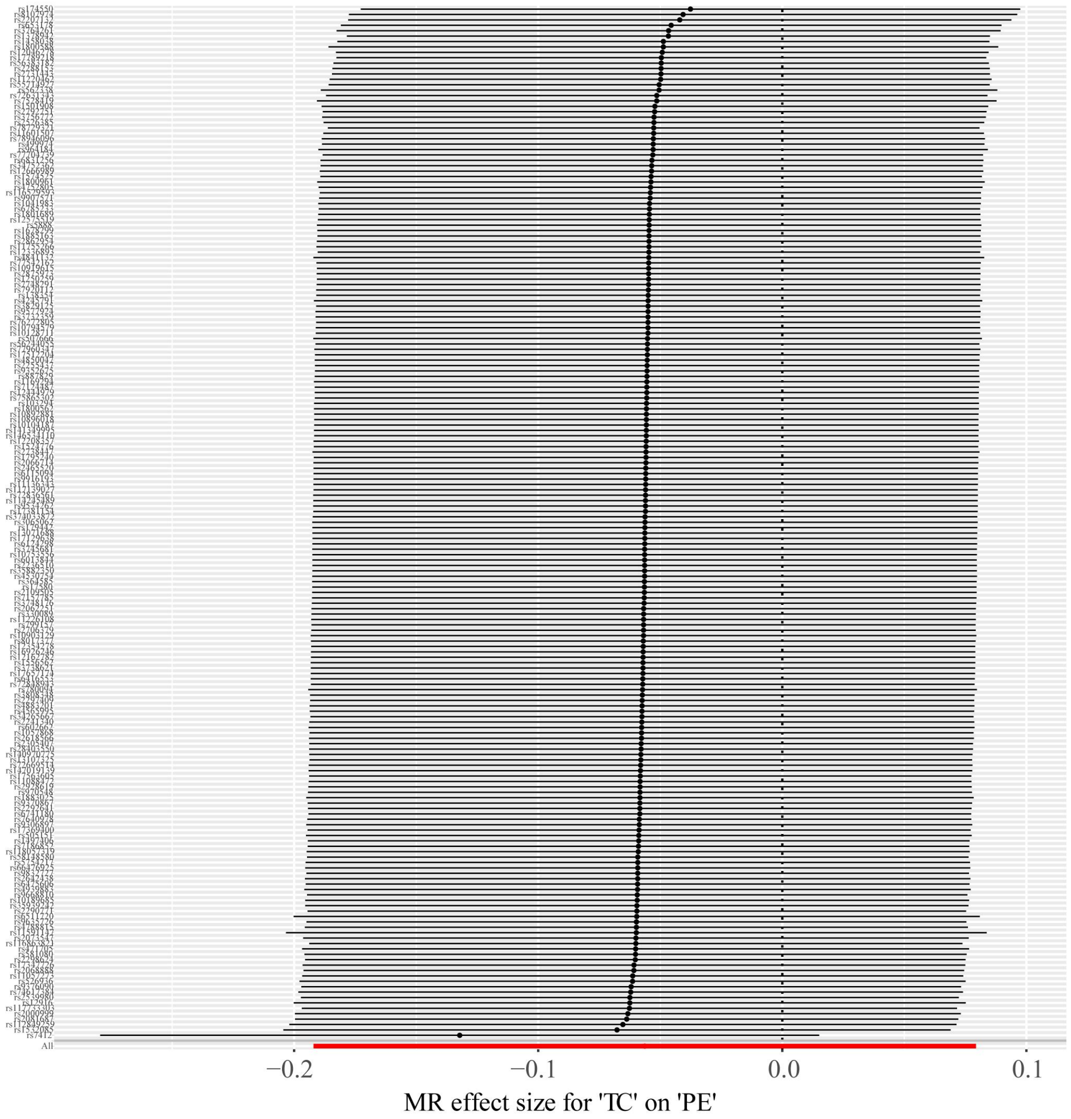

(G)

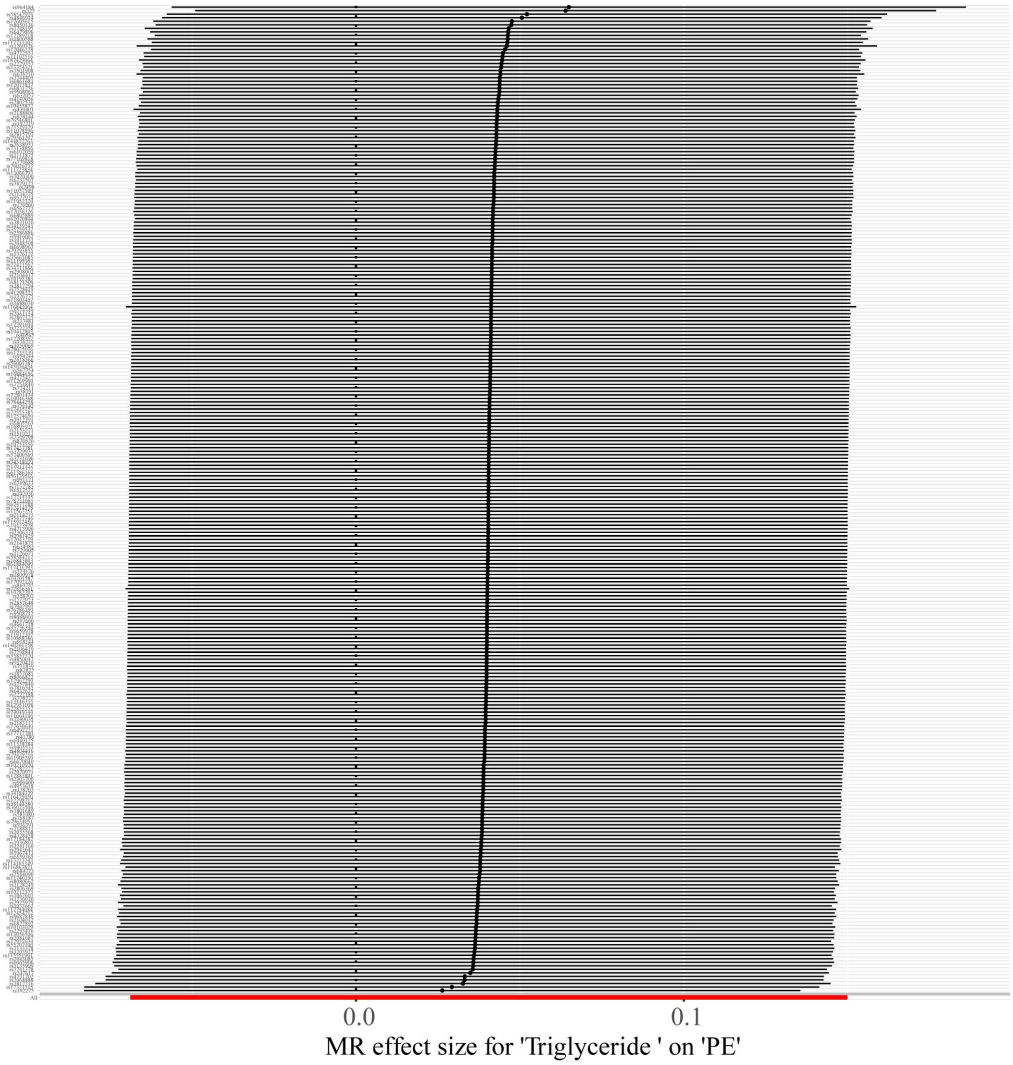

(H)

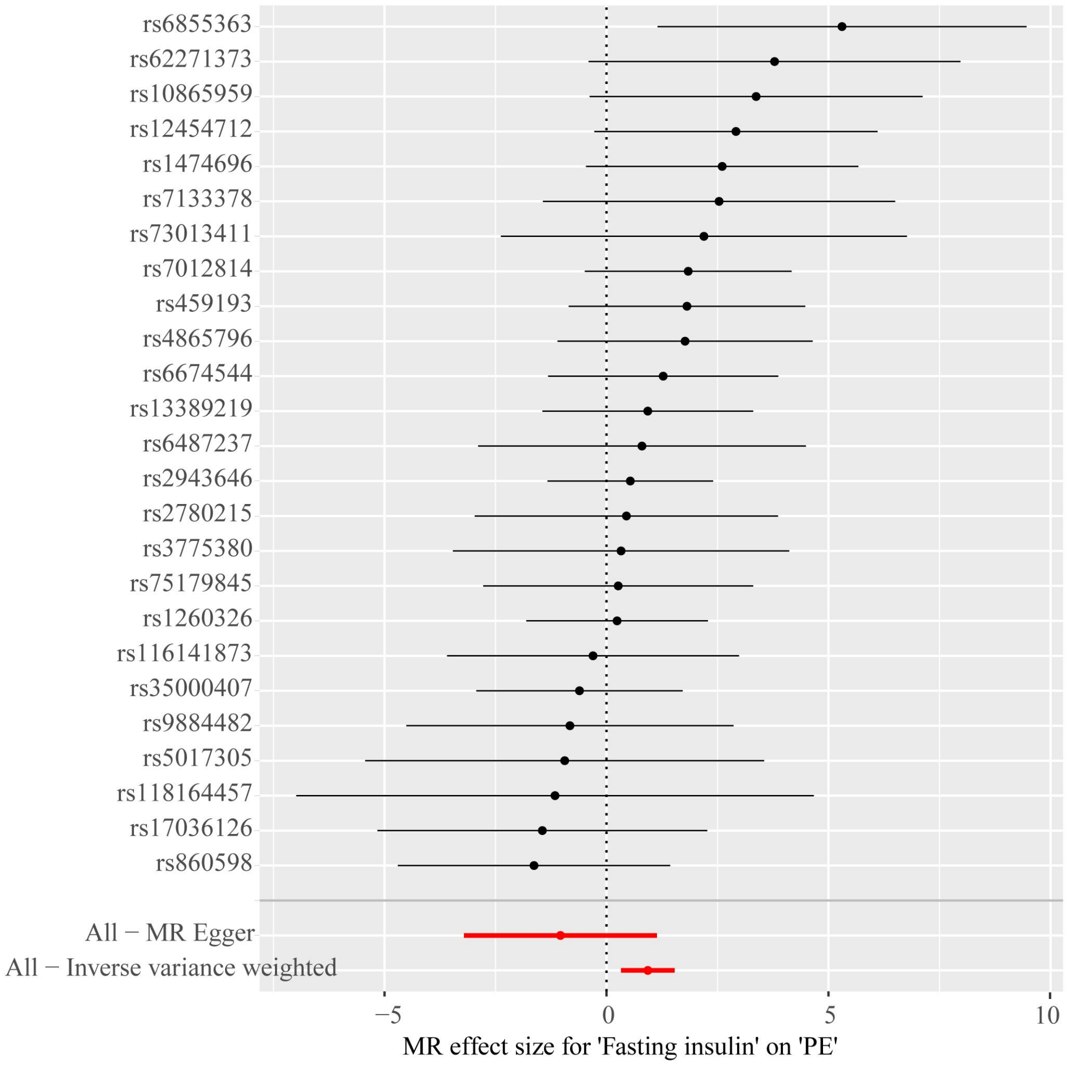
rs6855363
rs62271373
rs10865959
rs12454712
rs1474696
rs7133378
rs73013411
rs7012814
rs459193
rs4865796
rs6674544
rs13389219
rs6487237
rs2943646
rs2780215
rs3775380
rs75179845
rs1260326
rs116141873
rs35000407
rs9884482
rs5017305
rs118164457
rs17036126
rs860598
All − MR Egger
All − Inverse variance weighted
−5
0
5
10
MR effect size for 'Fasting insulin' on 'PE'

(I)

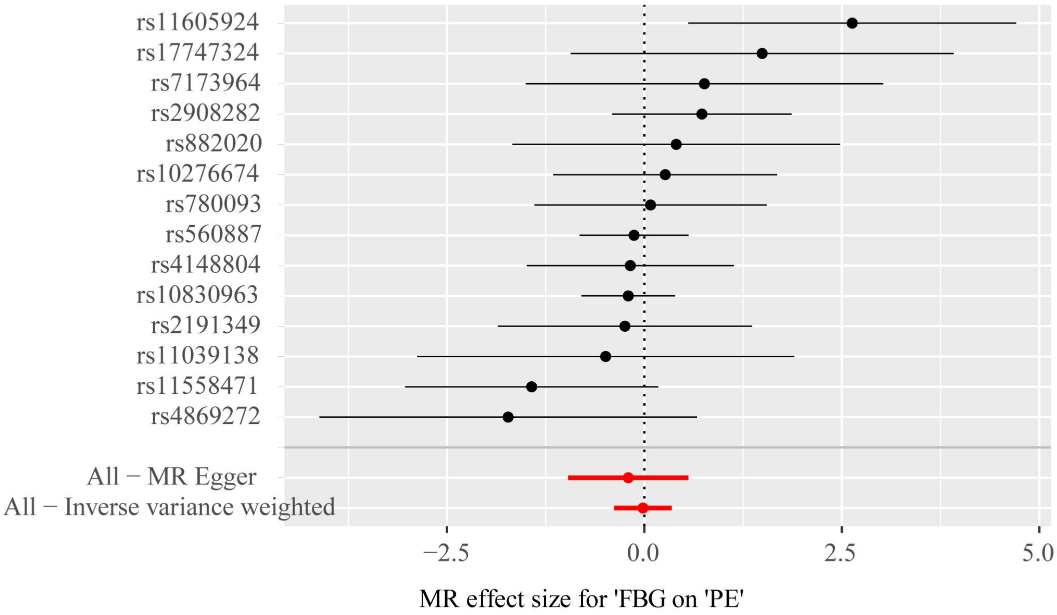

Supplementary Figures 2. Forest plots for the association of PCOS characteristic indicators associated SNPs with risk of PE. (A) T, (B) SHBG, (C) BMI ,(D) HDL,(E) LDL,(F) TC,(G) TG,(H) Fasting insulin,(I) Fasting blood glucose.HDL,high-density lipoprotein;LDL,Low density lipoprotein;BMI,body mass index;T, testosterone; SHBG, sex hormone binding globulin;TG,Triglyceride;TC,total cholesterol;PCOS Polycystic ovary syndrome; PE, preeclampsia; SNP, single-nucleotide polymorphism.
