# Supplementary material for: Genetically Predicted Causal Relationship Between Polycystic Ovary Syndrome and Preeclampsia
Source: Int J Endocrinol. 2026 Jul 14;2026:5536687. doi: 10.1155/ije/5536687 (PMC13366495; doi:10.1155/ije/5536687)
Supplement: Supplementary file 6 — Supporting Information 6 Supporting Figure 3. Leave‐one‐out plots for the causal associations between PCOS‐related traits and PE. [file IJE-2026-5536687-s006.pdf]

(A)

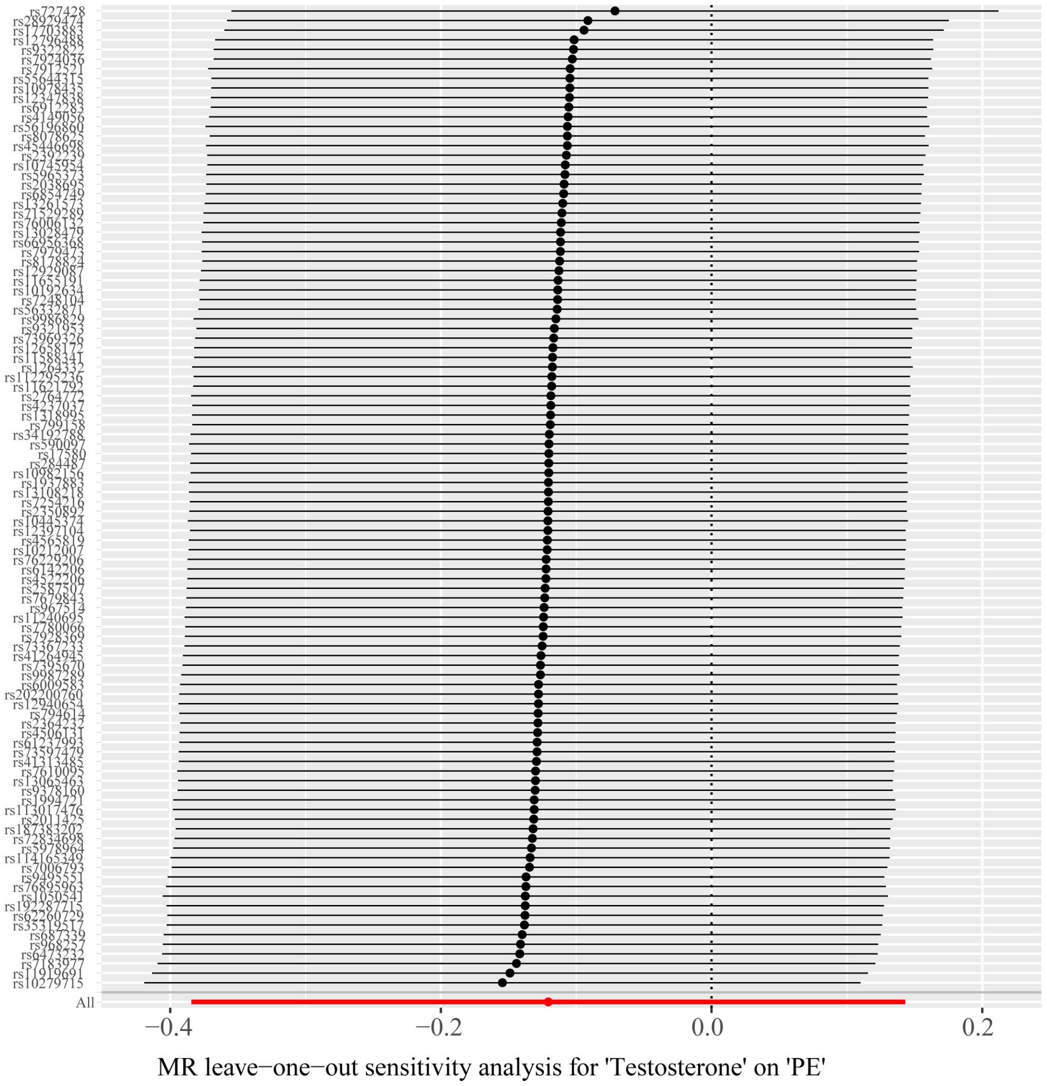
All
−0.4
−0.2
0.0
0.2
MR leave−one−out sensitivity analysis for 'Testosterone' on 'PE'

(B)

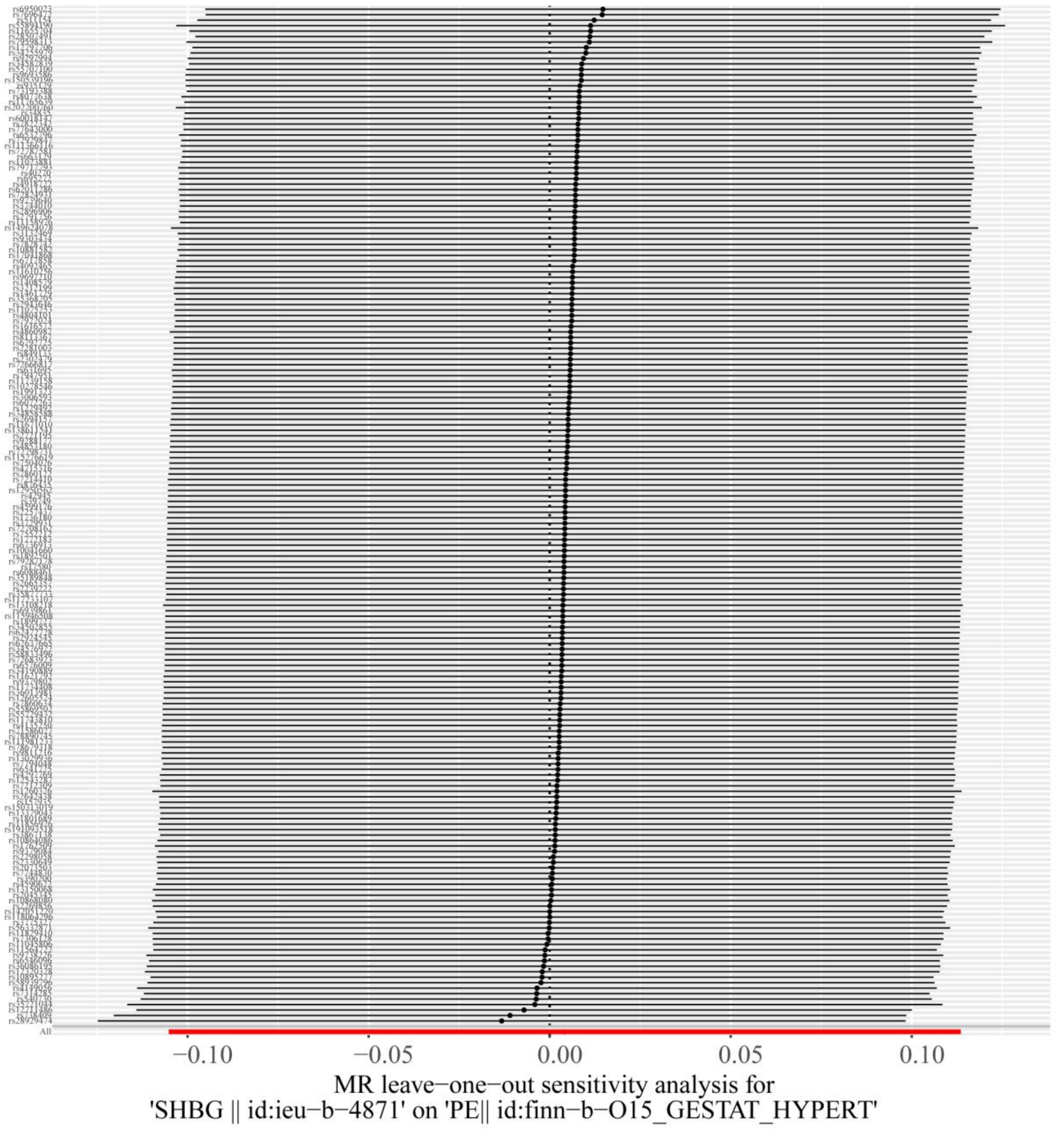
All
−0.10
−0.05
0.00
0.05
0.10
MR leave−one−out sensitivity analysis for
'SHBG || id:ieu−b−4871' on 'PE|| id:finn−b−O15_GESTAT_HYPERT'

(C)

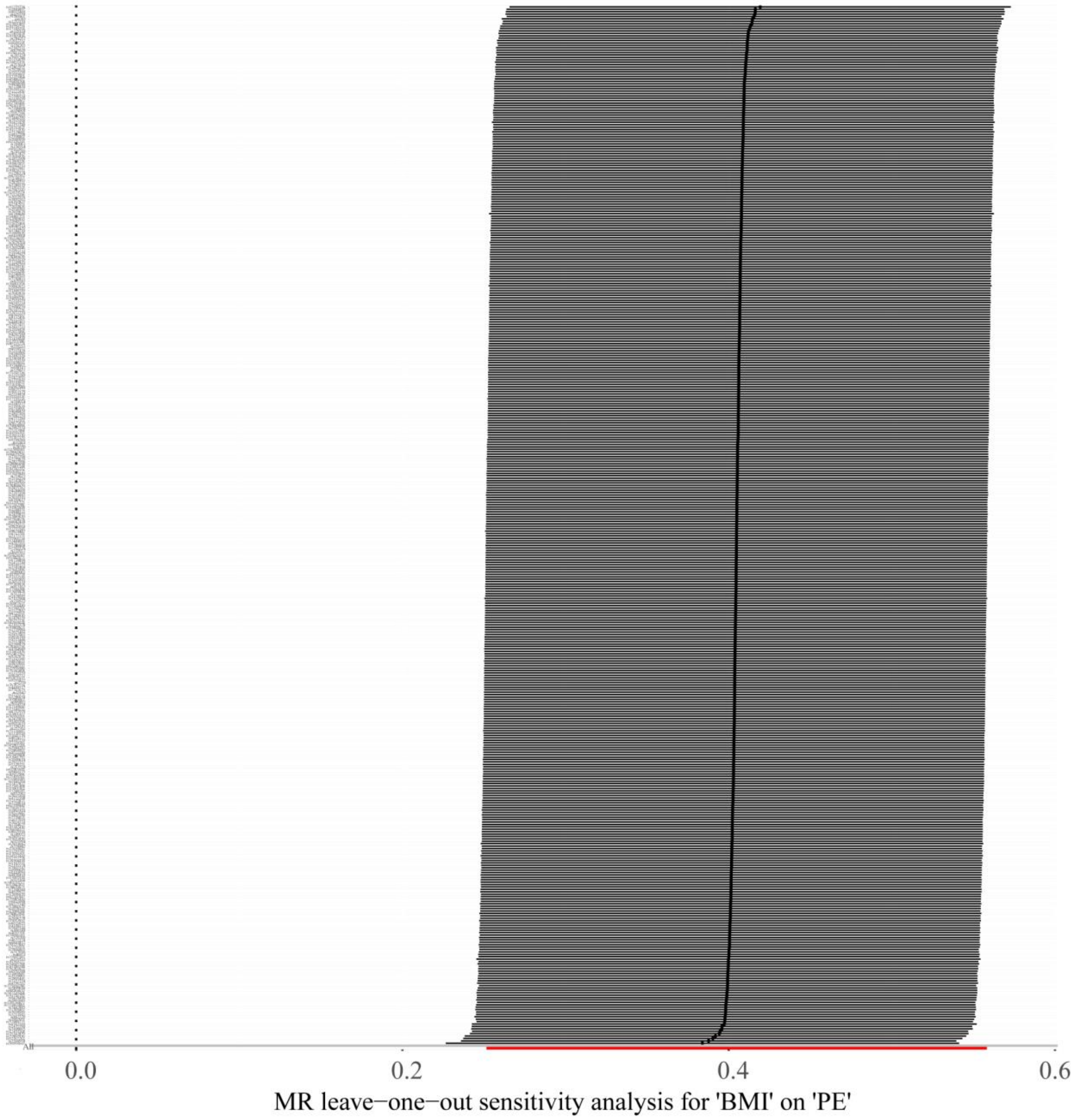
All
0.0
0.2
0.4
0.6
MR leave−one−out sensitivity analysis for 'BMI' on 'PE'

(D)

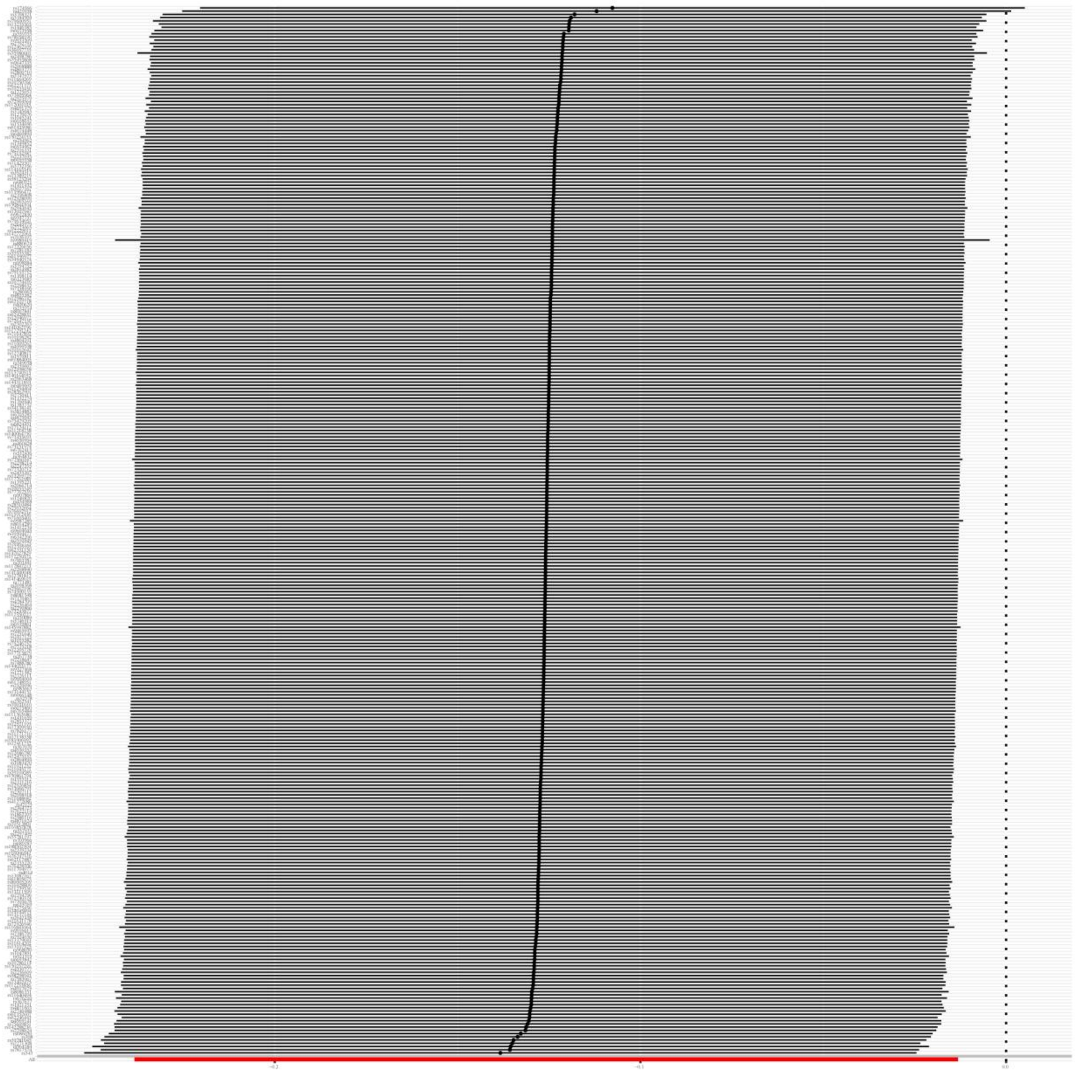

MR leave−one−out sensitivity analysis for 'HDL' on 'PE'

(E)

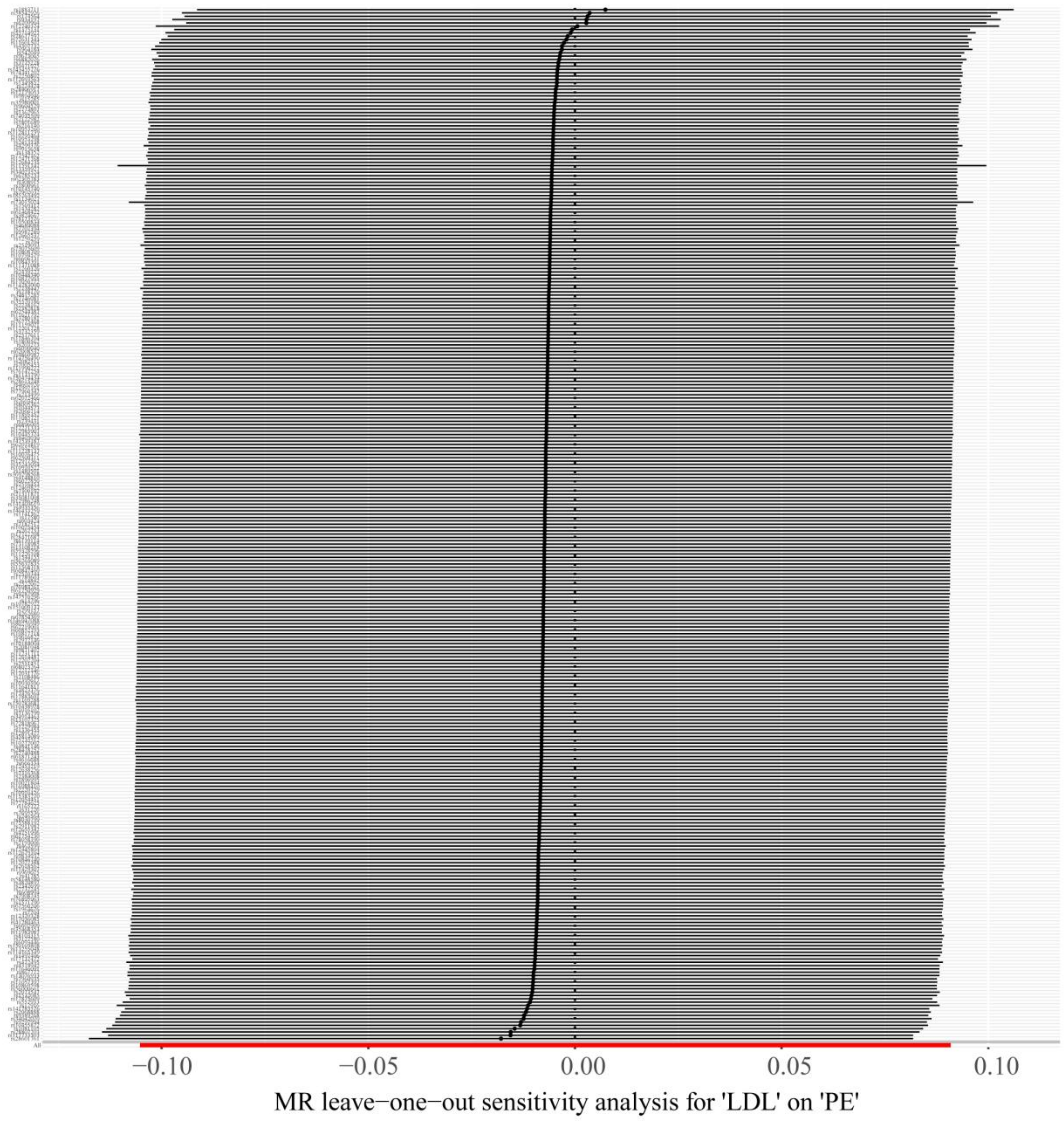

All
−0.10
−0.05
0.00
0.05
0.10
MR leave−one−out sensitivity analysis for 'LDL' on 'PE'

(F)

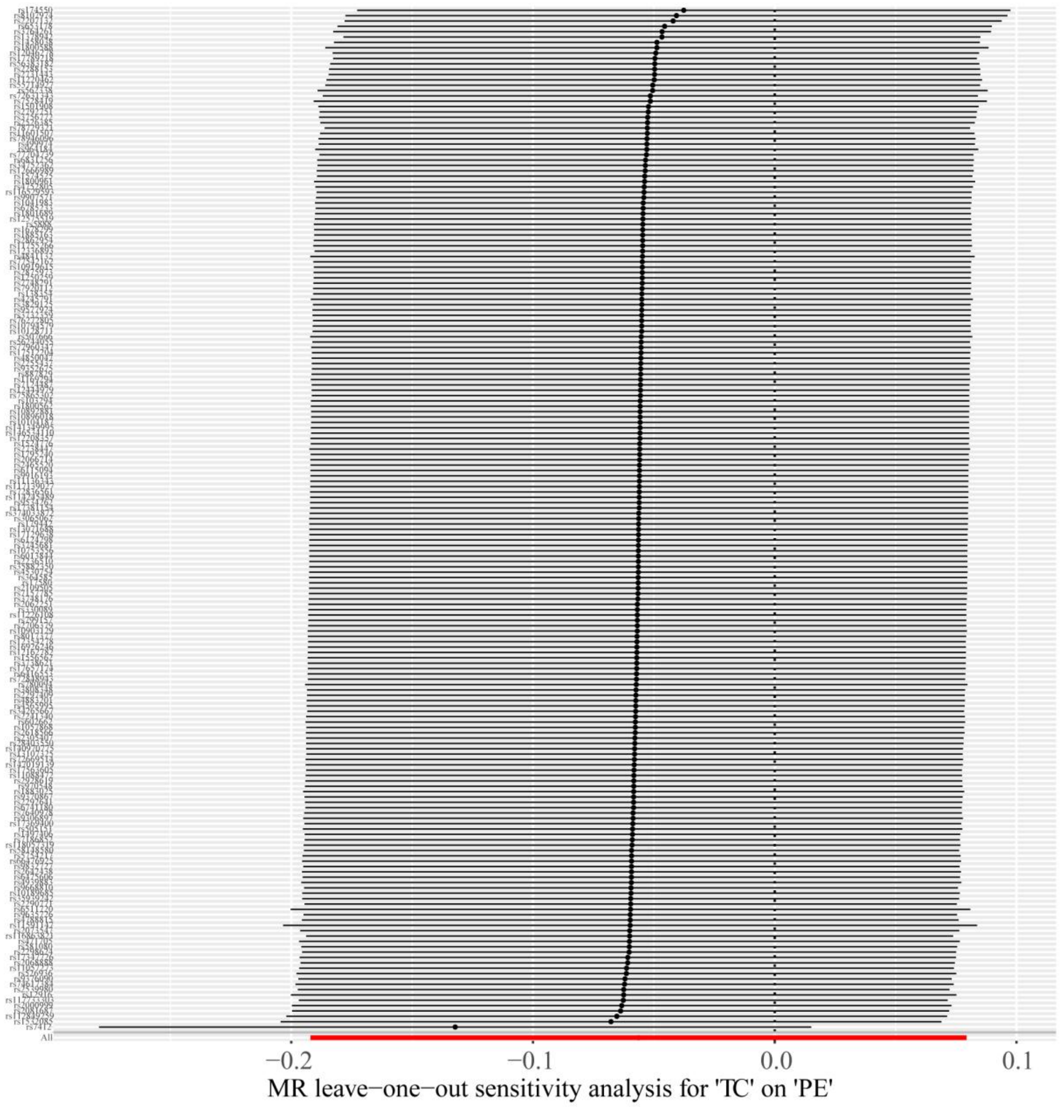
All
−0.2
−0.1
0.0
0.1
MR leave−one−out sensitivity analysis for 'TC' on 'PE'

(G)

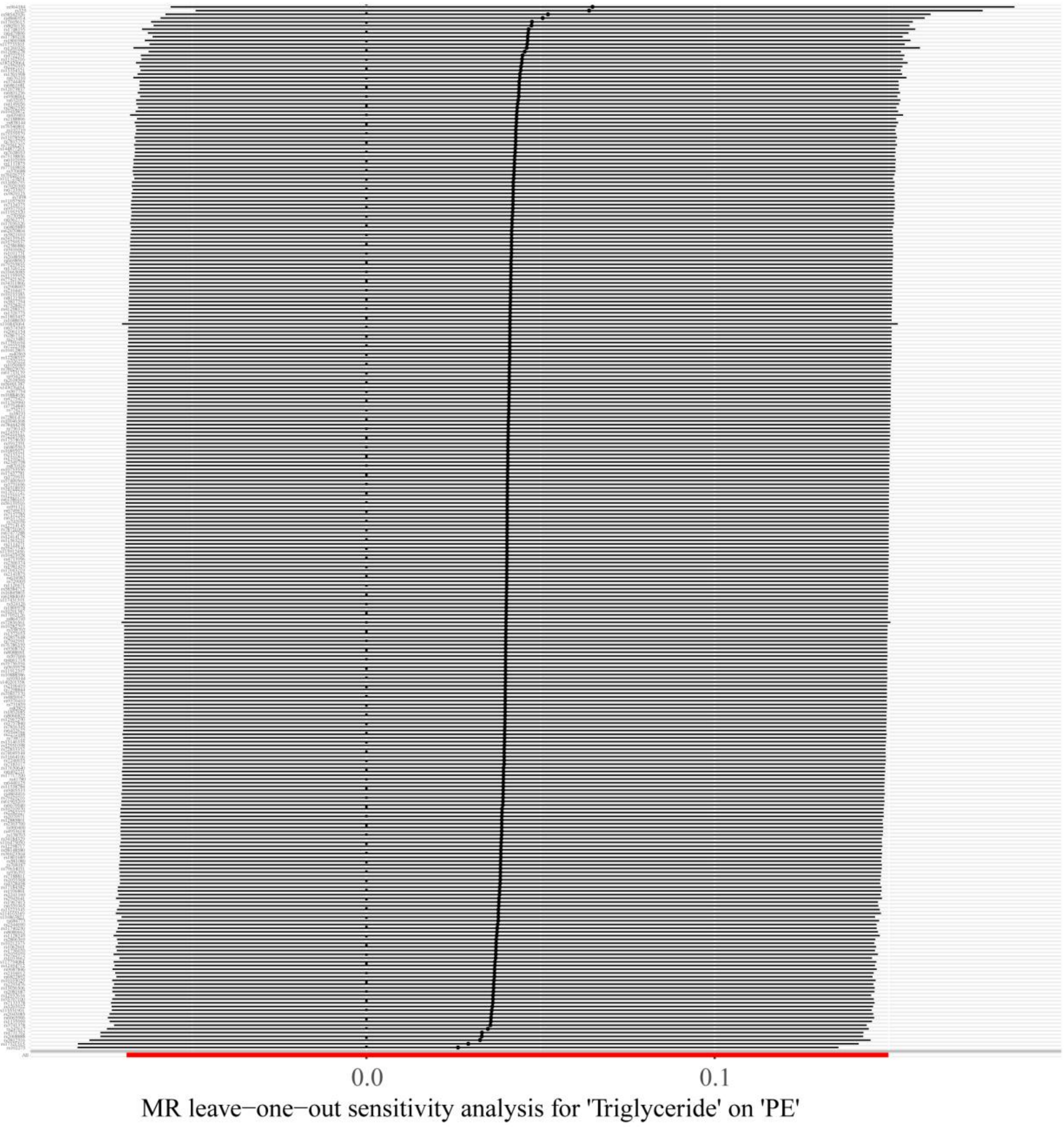
0.0
0.1
MR leave−one−out sensitivity analysis for 'Triglyceride' on 'PE'

(H)

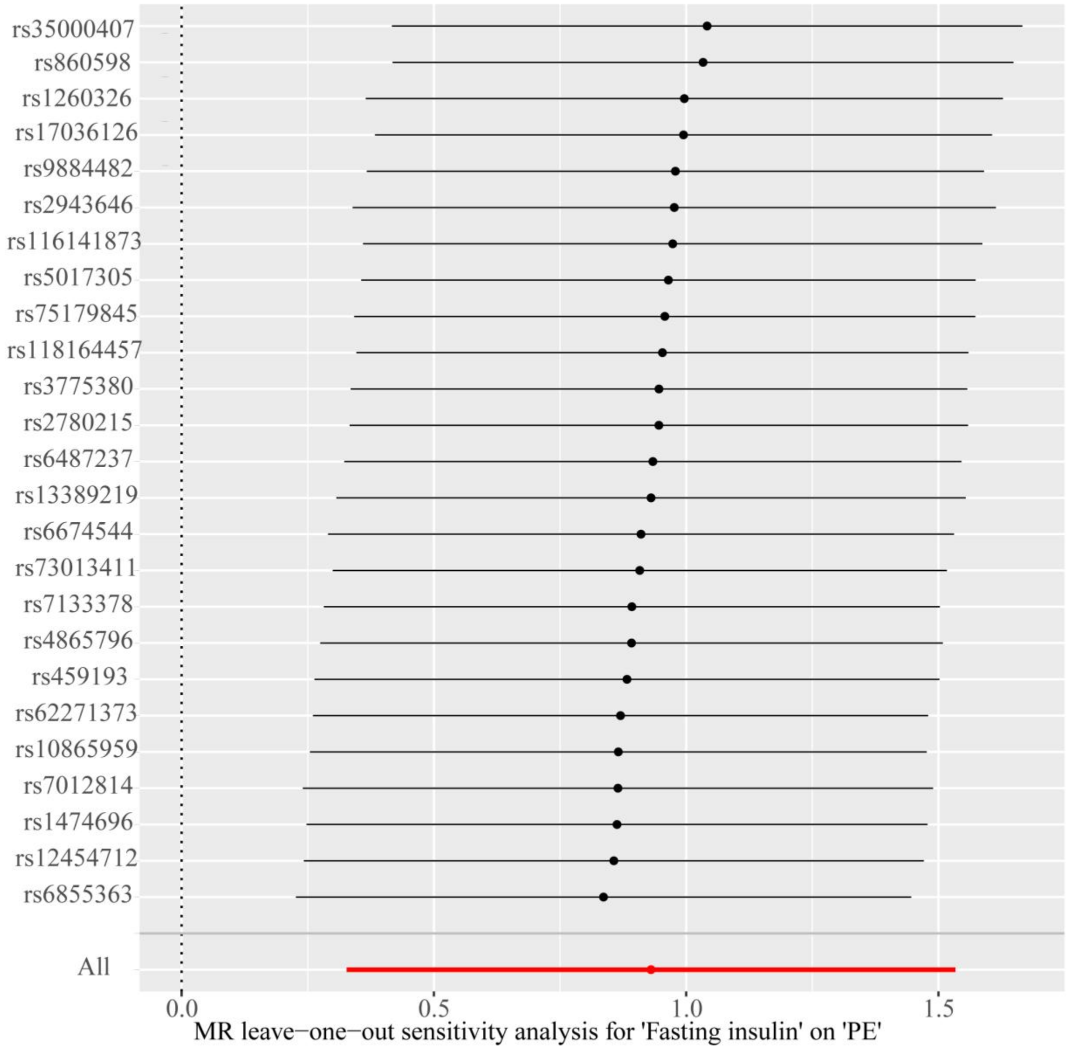
rs35000407
rs860598
rs1260326
rs17036126
rs9884482
rs2943646
rs116141873
rs5017305
rs75179845
rs118164457
rs3775380
rs2780215
rs6487237
rs13389219
rs6674544
rs73013411
rs7133378
rs4865796
rs459193
rs62271373
rs10865959
rs7012814
rs1474696
rs12454712
rs6855363
All
0.0
0.5
1.0
1.5
MR leave−one−out sensitivity analysis for 'Fasting insulin' on 'PE'

(I)

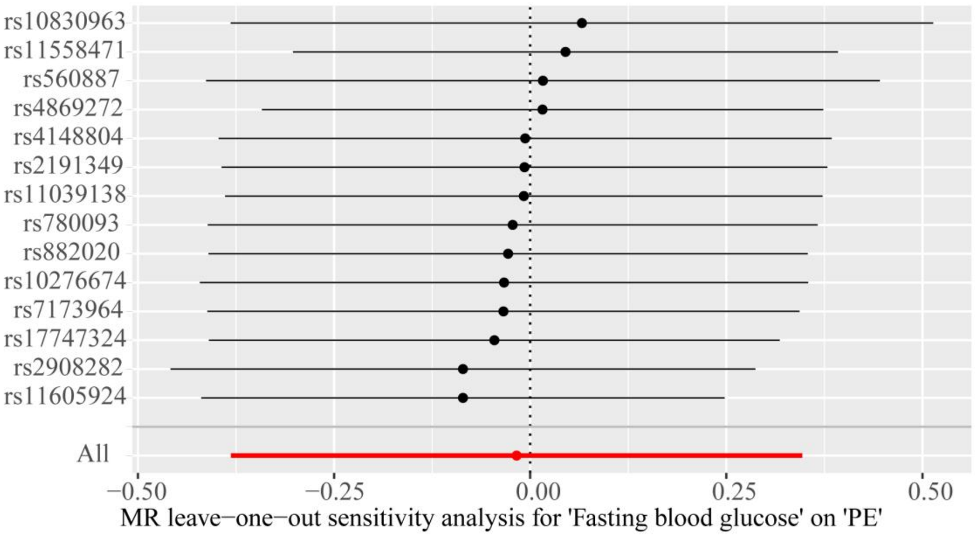

Supplementary Figures 3. Leave-one-out plots for the association of PCOS characteristic indicators associated SNPs with risk of PE. (A) T, (B) SHBG, (C) BMI ,(D) HDL,(E) LDL,(F) TC,(G) TG,(H) Fasting insulin,(I) Fasting blood glucose.HDL,high-density lipoprotein;LDL,Low density lipoprotein;BMI,body mass index;T, testosterone; SHBG, sex hormone binding globulin;TG,Triglyceride;TC,total cholesterol;PCOS Polycystic ovary syndrome; PE, preeclampsia; SNP, single-nucleotide polymorphism.
